# GATA binding protein 1 recruits histone deacetylase 2 to the promoter region of nuclear receptor binding protein 2 to affect the tumor microenvironment and malignancy of thyroid carcinoma

**DOI:** 10.1080/21655979.2022.2068921

**Published:** 2022-05-01

**Authors:** Mengyuan Li, Hongwei Jiang, Shengjiang Chen, Yujin Ma

**Affiliations:** a Department of Ultrasound, The First Affiliated Hospital, and College of Clinical Medicine of Henan University of Science and Technology, Luoyang, P.R. China; b Department of Endocrinology and Metabolism, The First Affiliated Hospital, and College of Clinical Medicine of Henan University of Science and Technology, Luoyang, P.R. China

**Keywords:** Thyroid carcinoma, tumor microenvironment, epigenetic regulation, *GATA1*, *HDAC2*, *NRBP2*

## Abstract

The tumor microenvironment (TME) and activated angiogenesis in thyroid carcinoma (TC) are critical for tumor growth and metastasis. Nuclear receptor binding protein 2 (*NRBP2*) has been suggested as a tumor suppressor. This study examines the function of *NRBP2* in the progression of TC and the regulatory mechanism. By analyzing bioinformatic tools including GSE165724 dataset and the Cancer Genome Atlas system, we predicted *NRBP2* as a poorly expressed gene in TC. Decreased *NRBP2* expression was detected in TC tumor tissues and cells. Poor expression of *NRBP2* was linked to unfavorable prognosis of patients. GATA binding protein 1 (*GATA1*) was found as a negative regulator of *NRBP2*. It recruited histone deacetylase2 (*HDAC2*) to the *NRBP2* promoter to trigger histone deacetylation. *NRBP2* overexpression suppressed growth of TC cells, and it reduced expression of TME markers, M2 polarization of macrophages, and angiogenesis in TC. Similar results were reproduced *in vivo* in nude mice. However, the anti-oncogenic roles of *NRBP2* were blocked after further overexpression of *GATA1* or *HDAC2*. In summary, this study demonstrates that *GATA1* recruits *HDAC2* to the *NRBP2* promoter and enhances the TME and angiogenesis in TC cells.

## GRAPHICAL ABSTRACT

**Figure:**
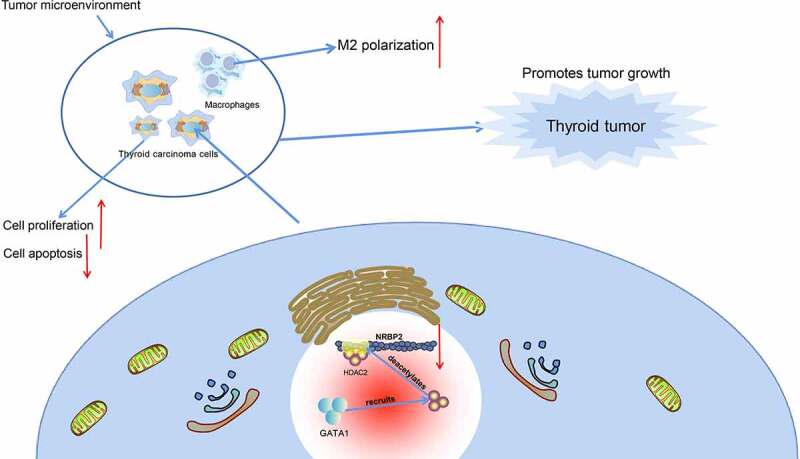

## Highlights

- Poor expression of NRBP2 in TC cells indicates poor prognosis in patients.
- NRBP2 reduces expression of TME markers and angiogenesis in TC cells.
- NRBP2 reduces TC tumorigenesis and M2 macrophage infiltration in vivo.
- GATA1 recruits HDAC2 to suppress NRBP2 expression through reducing H3K9ac level.
- GATA1 or HDAC2 blocks the inhibitory effect of oe-NRBP2 on TC cells.

## Introduction

Thyroid carcinoma (TC or THCA) represents the ninth most common cancer among all cancer types [1] and the most prevalent endocrine cancer as a neoplasm of the thyroid epithelium [2]. Ionizing radiation, especially in childhood, is the only well-recognized risk factor of TC [1]. Papillary TC (PTC), arising from the follicular cells, is the most frequent type that makes up about 80% of all cases [2]. The prognosis of patients with well-differentiated PTC was favorable with the 5-year survival rate reaching 97.5%, but the poorly differentiated types and anaplastic carcinomas are aggressive and lethal [3–5]. Tumor microenvironment (TME) plays a critical role in tumor migration and invasion [6]. Identifying novel molecules involved in TME maintenance and TC development may provide new biomarkers for improved risk prediction and help develop therapeutic options.

Advanced bioinformatic analytical systems have provided considerable benefits for researchers to identify crucial molecules implicated in the progression of human diseases including cancers [7,8]. In the present study, the microarray analysis using the TC-related gene expressing dataset GSE165724 from Gene Expression Omnibus (GEO; https://www.ncbi.nlm.nih.gov/geo/) suggested that nuclear receptor binding protein 2 (*NRBP2*) is significantly downregulated in TC tissues. The *NRBP* family initially participate in the transport between the endoplasmic reticulum and Golgi [9]. Moreover, the *NRBPs* are suggested to play tumor suppressive role consistently [10]. However, the function of *NRBP2* in the progression of TC remains not clear.

Gene alterations by genetical and epigenetic regulations are frequently involved in almost every stage of cancers including TC [11]. In this work, the bioinformatics analysis using JASPAR (http://jaspar.genereg.net/) suggested GATA binding protein 1 (*GATA1*) as a candidate negative regulator of *NRBP2. GATA1* is a master transcription factor in erythropoiesis which exert key functions in regulating proliferation, differentiation, and death of erythroid cells [12]. Recent evidence suggested the oncogenic role of *GATA1* in human malignancies [13,14]. Epigenetic mechanisms such as acetylation and methylation modifications that control transcriptional dysregulation in cancer development have aroused increasing concerns [15]. Overexpression of *GATA1* has been observed to interact with the histone methyltransferase *SET7* and to augment vascular endothelial growth factor (*VEGF*)-induced angiogenesis [16]. Interestingly, data in the UCSC browser (https://genome.ucsc.edu/index.html) demonstrated that there is significant acetylation of histone H3 lysine 9 (H3K9ac) in the promoter region of *NRBP2*. The histone H3 lysine 9 (H3K9) is a widely studied acetylation site producing H3K9ac, which is essentially correlated with transcriptional activation in cells [17]. However, as mentioned above, *NRBP2* was predicted to be poorly expressed in TC. Could this be caused by reduced H3K9ac level in the promoter of *NRBP2*? We therefore hypothesized that *GATA1* possibly interacts with specific histone deacetylases (*HDACs*) to reduce transcriptional activity of *NRBP2*, which participates in the progression of TC.

## Materials and methods

### Clinical samples

From February 2016 to October 2017, 71 patients with TC (27–81 years old, male: female = 2:5) admitted into the First Affiliated Hospital of Henan University of Science and Technology were enrolled into this research. The patients had low echogenicity, calcification and unclear border on the interior of the nodule by transthyroid ultrasound. According to the ACR Thyroid Imaging, Reporting and Data System (TI-RADS) scores [18], all patients were categorized in the TR3-TR5 grade, and they were diagnosed as TC according to the pathologic diagnosis. The fresh TC tumor tissues and the para-tumorous tissues were harvested during surgery and instantly frozen in liquid nitrogen. The research was approved by the Ethical Committee of the First Affiliated Hospital of Henan University of Science and Technology (Approval No. 2015.12.15) and adhered to the *Declaration of Helsinki*. Each participant signed the informed consent form.

### Bioinformatic analysis

The TC-related GEO dataset GSE165724 comprises 16 TC tissues and 12 healthy control tissues. Genes with differential expression between tumor and normal tissues were identified with Log Fold Change < −2 and adjusted *p* value < 0.01 as the screening thresholds. The data were analyzed using an R limma Package, and the heatmap was produced using the R Volcano package.

### Cell culture

A normal thyroid cell line Nthy-ori 3–1 (BNCC340487), two PTC cell lines BCPAP (BNCC358025) and TPC-1 (BNCC337912), and an undifferentiated thyroid squamous cell carcinoma cell line SW579 (BNCC100182) were procured from BeNa Culture Collection (Henan, China). Another undifferentiated TC cell line CAL62 (CL-0618) was procured from Procell Life Science & Technology Co., Ltd. (Wuhan, Hubei, China). After recovery, the cells were cultured in Roswell Park Memorial Institute (RPMI)-1640 containing 10% fetal bovine serum (FBS) at 37°C with 5% CO_2_. When a 90% confluence was reached, the cells were digested in 0.25% trypsin and sub-cultured at 1:3.

### Cell transfection

Overexpression vectors of NRBP2, HDAC2 and GATA1 (oe-NRBP2, oe-HDAC2 and oe-GATA1), the short hairpin (sh) RNA of NRBP2 and GATA1 (sh-NRBP2 and sh-GATA1), and the negative control (NC) vectors (oe-NC and sh-NC) were respectively transfected into TPC-1 and CAL62 cells using the pCMV6-AC-GFP vector (FH1215, Fenghui Biotechnology, Hunan, China). The overexpression plasmids and shRNA fragments were procured from Sigma-Aldrich Chemical Company (Merck KGaA, Darmstadt, Germany). In short, the target fragments and vectors were digested overnight at 37°C and then ligated with T4 DNA lignase. After that, 10 μL ligation product was added into 100 μL DH5α competent cells (TianGen Biotech Co., Ltd., Beijing, China). After concentration and centrifugation, the positive colony was identified by colony PCR. The vectors were extracted using a GenElute™ plasmid miniprep kit (PLN350, Sigma-Aldrich). Cell transfection was performed utilizing a Lipofectamine 2000 kit (11668019; Invitrogen, Thermo Fisher Scientific Inc., Waltham, MA, USA). In brief, 1 × 10^5^ cells were incubated in 24-well plates for 24 h before transfection. Thereafter, 1 μL Lipofectamine 2000 was added to the centrifugation tube with serum-free RPMI 1640 and mixed to prepare 25 μL diluted transfection reagent at a concentration of 25 nM. The vector solution for transfection and the dilution were loaded into centrifugation tubes and allowed to stand for 15 min until complete mixing. After that, the transfection compounds were loaded into cells in 0.45 mL medium for 6 h, and the cells were cultured in RPMI-1640 for 48 h. The transient transfection efficiency was identified by reverse transcription quantitative polymerase-chain reaction (RT-qPCR). Stably transfected cells were screened after culture with 1 μg/mL puromycin for 48 h. Later, the cells were harvested and the transfection efficacy was determined.

### RT-qPCR

Total RNA was extracted using the TRIzol reagent (15596018, Invitrogen). The RNA concentration was examined by a NanoDrop^TM^ Lite Spectrophotometer (Thermo Fisher Scientific), and a TaqMan PrimeScript RT kit (RR047A, Takara Holdings Inc., Kyoto, Japan) was used for RNA reverse transcription. After that, qPCR was conducted on an ABI 7500 qPCR system (Applied Biosystems, Inc., Carlsbad, CA, USA). Table 1 lists the sequence information of primers. Glyceraldehyde-3-phosphate dehydrogenase (GAPDH) was used as an internal loading. Gene expression value was analyzed using the 2^−ΔΔCt^ method [19].

**Table 1.** Primers for RT-qPCR

Gene Symbol	Forward (5ʹ-3ʹ)	Reverse (3ʹ −5ʹ)
NRBP2	GAGCCCTTTGACTCTGAGACCA	TTCCAGCACCAGAAGCAGAGTG
GATA1	CACGACACTGTGGCGGAGAAAT	TTCCAGATGCCTTGCGGTTTCG
HDAC2	CTCATGCACCTGGTGTCCAGAT	GCTATCCGCTTGTCTGATGCTC
GAPDH	GTCTCCTCTGACTTCAACAGCG	ACCACCCTGTTGCTGTAGCCAA

RT-qPCR, reverse transcription quantitative polymerase chain reaction; NRBP2, nuclear receptor binding protein 2; GATA1, GATA binding protein 1; HDAC2, histone deacetylase 2; GAPDH, glyceraldehyde-3-phosphate dehydrogenase.

### Western blot analysis

Total protein from cells was isolated using the radio-immunoprecipitation assay lysis buffer (P0013C, Beyotime Biotechnology Co., Ltd., Shanghai, China) containing phenylmethylsulfonyl fluoride. After concentration examination by a bicinchoninic acid kit (Thermo Fisher Scientific), an equal amount of protein sample (50 μg) was separated by 12% sodium dodecyl sulfate-polyacrylamide gel electrophoresis (SDS-PAGE) and loaded onto polyvinylidene fluoride membranes (Millipore Corp., Billerica, MA, USA). After that, the membranes were blocked by 5% nonfat milk for 1 h and reacted with the antibodies against NRBP2 (1:1,000, PA5-65039, Thermo Fisher Scientific), GATA1 (1:1,000, ab181544, Abcam Inc., Cambridge, MA, USA) and GAPDH (1:2,500, ab181602, Abcam) at 4°C overnight, and then with goat anti-rabbit immunoglobulin G (IgG) (1:2,000, ab97051, Abcam) at 22–25°C for 1 h. The protein blots were visualized using an enhanced chemiluminescence kit (P0018FS; Beyotime Biotechnology Co., Ltd., Shanghai, China) and photographed by an image analyzing system (Bio-Rad Laboratories, CA, USA). The intensity of the protein signals was determined using the Quantum One v.4.6.2 software with GAPDH as the internal loading [20].

### 5-ethynyl-2’-deoxyuridine (EdU) labeling assay

Cell proliferation was analyzed using the EdU kit (C10310-2, RiboBio Co., Ltd., Guangzhou, Guangdong China). In short, transfected cells were cultured in 12-well plates for 36 h. After that, the cells were cultured in serum-free medium containing EdU (50 μM) for 2 h, washed with phosphate-buffered saline, and fixed for 30 min. After that, the cells were stained with Apollo and Hoechst 33,342 in the dark for 30 min. The staining images were captured under a microscope (Olympus, Tokyo, Japan) with five random fields of views included [21].

### Colony formation assay

Exponentially growing TPC-1 or CAL-62 cells were adjusted to 10^3^ cells/mL in the culture medium. After that, 1.5 mL cell suspension was seeded in culture dishes mixed with 5% agar and culture medium at a ratio of 1:9. The cells were cultured at 37°C with 5% CO_2_ for 2 weeks. After that, the cells were fixed for 30 min and stained with 0.1% crystal violet for 3 min. The colonies were observed and counted under microscopy [22].

### Enzyme-linked immunosorbent assay (ELISA)

The interleukin (IL)-6 and vascular endothelial growth factor A (VEGFA) secreted by TPC-1 or CAL62 cells were examined using the IL-6 (#D6050, R&D Systems, Minneapolis, MN, USA) and VEGFA (#DVE00, R&D system) ELISA kits following the manufacturer’s instruction manual.

### Co-culture with macrophages

Transfected TPC-1 and CAL62 cells were loaded into the Transwell upper wells. Wells filled with RPMI-1640 were set as controls. THP-1 cells (American Type Culture Collection, Manassas, VA, USA) were induced with phorbol-12-myristate-13-acetate (PMA; Sigma-Aldrich) to obtain the phenotype of M0 macrophages, which were loaded into the basolateral chambers. After 48 h, relative mRNA expression of CD206 and CD163 (macrophage biomarkers) in the macrophages was examined using RT-qPCR. The percentage of CD206/CD163-positive cells was examined using flow cytometry [23].

### Immunofluorescence staining

M2 polarization of macrophages was detected by immunofluorescence staining of Arginase-1 (Arg1). The macrophages were incubated with anti-Arg1 (1:300, #93668, Cell Signaling Technology (CST), Beverly, MA, USA) and then with the secondary antibody (1:1,000, ab150077, Abcam) at 25°C for 1 h. Later, he cells were counterstained with 10 mg/mL 4’, 6-diamidino-2-phenylindole. The staining was analyzed under a fluorescence microscope [24].

### Tube formation assay

Human umbilical vein endothelial cells (HUVECs) (Yiyuan Biotechnology Corporation, Guangzhou, Guangdong, China) were cultured into Matrigel-coated 24-well plates at 2 × 10^4^ cells per well and co-cultured with the supernatant of TPC-1 and CAL62 cells. After 24 h, the number of vascular branches was examined under a phase-contrast microscope (Olympus) with 5 random fields included [24].

### Co-immunoprecipitation (Co-IP)

Before the collection of protein extracts, cells were dissolved in lysis buffer. The cell lysis buffer was mixed with Flag M2-affinity gel-conjugated GATA1 antibody at 4°C for 12 h. After that, the protein was gradually eluted by KCl-contained buffer. The lysates (approximately 300 µg protein) were incubated with fresh protein A-beads (35 µL, #9863, CST) and 1 µg anti-GATA1 (1:1,000, ab181544, Abcam), anti-Flag (1:500, AF519, Beyotime) or IgG (1:500, ab172730, Abcam) at 4°C for 3 h. The magnetic beads were lysed in lysis buffer and loaded in SDS-PAGE. The immunoprecipitated proteins were reacted with anti-HDAC1 (1:1,000, ab109411, Abcam), anti-HDAC2 (1:1,200, ab32117, Abcam) and anti-HDAC3 (1:2,000, ab32369, Abcam). The immunoblot reactions were performed as described previously [25].

### Flow cytometry

Flow cytometry was first applied to examine cell apoptosis. After transfection, the TC cells were detached in ethylene diamine tetraacetic acid-0.25% trypsin and collected into the tubes. The cells were centrifuged with the supernatant discarded. The Annexin V-fluorescein isothiocyanate (FITC)/ propidium iodide (PI) staining solution was prepared following instructions of the Annexin V-FITC/PI staining kits (K201-100, BioVision, Milpitas, CA, USA). The FITC, PI and 4-(2-hydroxyethyl)-1-piperazineethanesulfonic acid (HEPES) buffer (PB180325; Procell) were mixed at a 1:2:50. Every 1 × 10^6^ cells were resuspended in 100 μL staining solution and incubated at 22–25°C for 15 min. After that, the cells were further treated with 1 mL HEPES buffer. Apoptosis of cells was determined analyzed by the flow cytometer (Beckman Coulter, CA, USA) [26].

Polarization of macrophages was examined by flow cytometry as well. The PMA-treated THP-1 cells (macrophages) were co-cultured with cancer cells for 48 h (macrophages: cancer cells = 1:5). Thereafter, the M1 or M2 polarization of the macrophages was determined using the CD86-APC antibody (1:20, 374207, Biolegend, San Diego, CA, USA) and CD206 PE antibody (1:50, 566884, BD Biosciences, Franklin Lakes, NJ, USA).

### CRISPR-cas9 system

The single-guide RNA (sgRNA) of GATA1 and the NC were obtained from Sigma-Aldrich. The sgRNA was cloned to the Cas-9 plasmid using the restriction endonuclease. The ligation products were transformed into competent cells, which were cultured for 12 h for plasmid monoclonal amplification. Thereafter, the plasmids were screened and extracted, and then transfected into TPC1 and CAL26 cells to construct GATA1-deleted cells and the wild-type (WT) control cells. The primer sequences are as follows: GATA1-promoter-1#: Guide Sequence: GCCCCCATAAGCACTATTG, protospacer adjacent motif: GGG; GATA1-promoter-2#: Guide Sequence: CGCTTCTTGGGCCGGATGA, protospacer adjacent motif: GGG.

### Animal experiments

Twenty-four female BALB/c nude mice (4–5 weeks old, 15–18 g) were acquired from SLAC Laboratory Animal Co., Ltd. (Shanghai, China). The mice were allocated into four groups, n = 6 in each. TPC-1 or CAL62 cells transfected with oe-NRBP2 or oe-NC were injected into the mice subcutaneously. Thereafter, the volume (V) of xenograft tumors was examined once per week. On the 36^th^ day, the mice were sacrificed via intraperitoneal injection of overdosed barbiturate (120 mg/kg). The tumors were weighed and used for further analysis [27]. All animal experiments were approved by the Animal Ethics Committee of the First Affiliated Hospital, and College of Clinical Medicine of Henan University of Science and Technology (Approval No. 2019. 1.19) and adhered to the Guide for the Care and Use of Laboratory Animals (National Institutes of Health, Bethesda, Maryland, USA). Significant efforts were made to reduce the usage and suffering of conscious animals.

### Immunohistochemistry (IHC)

The xenograft tumor tissues were fixed, embedded, and cut into 5-μm slices. The slices were dewaxed, treated with streptavidin peroxidase. After 10 min of antigen retrieval, the slices were blocked by blocking reagent. IHC was conducted using a Histostain SP-9000 IHC kit (Invitrogen, Thermo Fisher Scientific). The slices were hybridized with anti-NRBP2 (1:100, GTX117169, GeneTex Inc., San Antonio, TX, USA) anti-KI67 (1:1,000, ab15580, Abcam), anti-Arg1 (1:100, #93,668, CST) and anti-CD31 (1:2,000, ab182981, Abcam) overnight at 4°C, and then with the goat anti-rabbit IgG (1:10,000, ab6721, Abcam) at 37°C for 30 min. The slices were thereafter incubated with HRP-labeled working solution at 22–25°C for 30 min and developed with 3,3’-diaminobenzidine. After that, the slices were counterstained with hematoxylin and sealed for microscopy observation [28].

### Chromatin immunoprecipitation (ChIP)-qPCR

A ChIP analysis kit (Cat#53008, Active Motif, Carlsbad, CA, USA) was used. The TC cells were crosslinked in 1% methanol for 10 min and neutralized with glycine for 5 min. After that, the cells were resuspended in SDS lysis buffer, ultrasonicated and centrifuged. The supernatant was diluted in IP dilution buffer. Anti-GATA1 (1:50, #3535, CST), anti-H3K9ac (1:100, GTX128944, Genetex) and IgG (1:1,000, Cat#2729S, CST) were used for IP reaction. Thereafter, the samples were added with Protein A-agarose for 1 h of incubation. The precipitates were washed and de-crosslinked, and the purified DNA was determined by qPCR [29]. The primer sequences are presented in Table 2.

**Table 2.** Primers for ChIP-qPCR

	Forward (5ʹ-3ʹ)	Reverse (5ʹ-3ʹ)
ChIP	GAGCCAGAGCAGAGCTTCC	AGGACGAGGAGGGGACA
Negative	GTCCTCCACACCAGAATC	GGAATAGGCTGCTGAATTG

ChIP-qPCR, chromatin immunoprecipitation-quantitative polymerase chain reaction

### Luciferase assay

The promoter sequence of *NRBP2* was obtained from UCSC and sub-cloned to the pGL3 luciferase vector (E1751, Promega, Fitchburg, WI, USA). To confirm the binding between *NRBP2* promoter and *GATA1* or *HDAC2*, the WT GATA1 or HDAC2 sequence was cloned into the pGL3 vector to construct pGL3-WT luciferase vectors. Sequence products were gene fragments containing motif-binding sequences, and product lengths were controlled to range from 500 to 600 bp. The putative-binding sequence between *NRBP2* promoter and *GATA1* was obtained from JASPAR, whereas its H3K9ac binding site with *HDAC2* was obtained for UCSC. After that, different doses of pGL3-GATA1-WT or pGL3-HDAC2-WT vectors were transfected with pCMV6-AC-GFP-based oe-GATA1 or oe-HDAC2 into 293 T cells. After 24 h, the cells were collected. The luciferase activity in cells was examined using a dual-luciferase reporter gene system (Promega) [30].

### Statistical analysis

Prism 8.01 (GraphPad, La Jolla, CA, USA) and SPSS20.0 (IBM Corp. Armonk, NY, USA) was used for data analysis. Data are presented as the mean ± standard deviation from three repetitions. Differences analyzed by *t* test, or by one- or two-way analysis of variance followed by Tukey’s post-hoc comparison. Correlations between gene expression and the clinical characteristics of patients with TC were analyzed by the Fisher’s exact test or the Chi-square test. *p < 0.05 represents significant difference.

## Results

### Starting paragraph

We obtained via bioinformatic analyses that *NRBP2* is a downregulated in gene TC tissues and *GATA1* a candidate negative regulator of *NRBP2*. With significant H3K9ac modification predicted in *NRBP2* promoter, we hypothesized that *GATA1* possibly interacts with specific HDACs to reduce transcriptional activity of *NRBP2*. To validate this, we examined the interactions between *GATA1, HDAC2,* and *NRBP2* via Co-IP and ChIP assays. Altered expression of *NRBP2, GATA1,* or *HDAC2* was introduced in TC cells to evaluate their functions in the malignant behaviors of cells and the TME markers. Xenograft tumors were introduced in nude mice for *in vivo* experiments.

### *Poor expression of* NRBP2 *in TC cells is linked to poor prognosis in patients*

**Figure 1.**
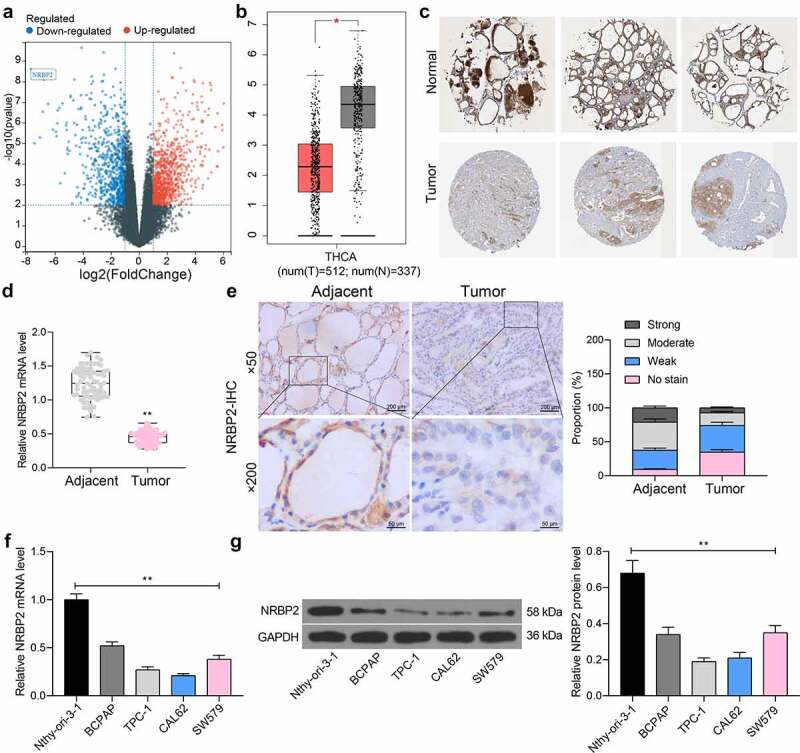
Poor expression of *NRBP2* in TC cells indicates unfavorable prognosis in patients. (a), differentially expressed genes between TC and normal samples in the GSE165724 dataset; (b), *NRBP2* expression predicted in TCGA-THCA; (c), IHC data of NRBP2 predicted via the HPA system; (d), mRNA expression of *NRBP2* in tissues from 71 TC patients examined by RT-qPCR; (e), representative IHC images of NRBP2 and staining intensity in tumor tissues (PTC) and the adjacent normal from patients; (f)-(g), mRNA (f) and protein (g) levels of NRBP2 in Nthy-ori 3–1 cells and TC cell lines (BCPAP, TPC-1, CAL62 and SW579) examined by RT-qPCR and western blot analysis. Three repetitions were performed. **p < 0.01.

The TC-related gene expression dataset GSE165724 comprising 16 TC tissues and 12 healthy control tissues was analyzed. By using Log Fold Change < −2 and adjusted *p* value < 0.01 as the screening thresholds, 834 genes with differential expression in TC tissues were screened (Figure 1(a)). These genes were compared with the data in The Cancer Genome Atlas-Thyroid Carcinoma (TCGA-THCA) (https://www.cancer.gov/types/thyroid), which suggested that *NRBP2* was significantly reduced in TC tissues (Figure 1(b)). Moreover, we obtained the IHC data of NRBP2 in normal thyroid tissues and TC tissues from the HUMAN PROTEIN ATLAS (HPA, https://www.proteinatlas.org/). It was suggested that the *NRBP2* expression is lower in tumor samples (moderate) than that in healthy samples (high) (Figure 1(c)). After that, we examined the expression of *NRBP2* mRNA in the 71 included patients using RT-qPCR and confirmed that *NRBP2* was expressed at low levels in the tumor tissues versus the adjacent tissues (adjacent tissue vs. tumor: 9.55 vs. 3.44 (all mean values, the same below); *p* < 0.0001) (Figure 1(d)). In addition, our IHC results showed that the IHC intensity of NRBP2 was quite weak in most tumor tissues, whereas the adjacent tissues had increased portion of moderate or strong staining intensity (adjacent tissue vs. tumor: No stain: 9.45 vs. 34.69, *p* < 0.0001; Weak: 28.17 vs. 29.44, *p* = 0.0032; Moderate: 41.25 vs. 18.47, *p* < 0.0001; Strong = 21.13 vs. 7.40, *p* < 0.0001) (Figure 1(e)). The relevance of *NRBP2* to the clinical characteristics of patients was further analyzed. It was found that *NRBP2* expression showed no significant relevance to the tumor size, sex, age, patients, or the histological type, whereas poor expression of *NRBP2* in patients was linked to increased infiltration depth, lymph node metastasis, poor tumor differentiation, and ACR TI-RADS scores (Tables 3–4). The NRBP2 expression in cells was examined thereafter. The RT-qPCR results showed that NRBP2 mRNA levels were decreased in the TC cell lines (BCPAP, TPC-1, CAL62 and SW579) versus the Nthy-ori 3–1 cells (Nthy-ori-3-1 vs. BCPAP/TPC-1/CAL62/SW579: 1.00 vs. 0.52/0.27/0.21/0.39; all *p* < 0.0001) (Figure 1(f)). Likewise, decreased protein levels of NRBP2 were detected in cancer cells (Nthy-ori-3-1 vs. BCPAP/TPC-1/CAL62/SW579: 0.68 vs. 0.34/0.19/0.21/0.31; all *p* < 0.0001) (Figure 1(g)).

**Table 3.** Correlation between NRBP2 expression and the clinical characteristics of patients

		NRBP2 expression		*p* value
Characteristics	Total (*n* = 71)	Low (*n* = 36)	High (*n* = 35)	
Age				0.809
>50	27	13	14	
<50	44	23	21	
Gender				0.594
Male	19	11	8	
Female	52	25	27	
Histological type				0.4953
Papillary thyroid carcinoma	53	29	24	
Follicular carcinoma of the thyroid	10	3	7	
Undifferentiated Thyroid carcinoma	5	3	2	
Medullary thyroid carcinoma	3	1	2	
Extrathyroid extension				0.0026
No or minimal	46	17	29	
Significant	25	19	6	
Lymph node metastasis				<0.0001
Negative	44	13	31	
Positive	27	23	4	
Tumor size				0.775
>3 cm	16	9	7	
<3 cm	55	27	28	
Differentiation level				0.299
Poor	21	13	8	
Moderate or Strong	50	23	27	
ACR TI-RADS score (%)				0.0003
TR3	20	4	16	
TR4	21	9	12	
TR5	30	23	7	

NRBP2, nuclear receptor binding protein 2; TI-RADS, Thyroid Imaging, Reporting and Data System; NA, not applicable. Correlations of NRBP2 expression with age, gender, extrathyroid extension, lymph node metastasis, tumor size and differentiation level of patients are analyzed by the Fisher’s exact test; correlations of NRBP2 expression with histological type and the ACR TI-RADS score of patients were analyzed by the Chi-square test.

**Table 4.** Clinical characteristics of included patients with TC

Number	Gender	NRBP2 expression	Histological type	Extrathyroid extension	Lymph node metastasis	ACR TI-RADS score (%)
1	Male	6.46	Papillary thyroid carcinoma	Significant	Positive	TR3
2	Female	6.8	Papillary thyroid carcinoma	Significant	Positive	TR4
3	Female	10.73	Papillary thyroid carcinoma	Minimal	Negative	TR5
4	Female	9.4	Undifferentiated Thyroid carcinoma	Significant	Positive	TR5
5	Female	10.44	Papillary thyroid carcinoma	Significant	Negative	TR3
6	Female	11.86	Papillary thyroid carcinoma	Minimal	Negative	TR5
7	Male	10.88	Follicular carcinoma of the thyroid	Minimal	Negative	TR4
8	Female	8.11	Papillary thyroid carcinoma	Significant	Positive	TR5
9	Male	8.93	Papillary thyroid carcinoma	Significant	Negative	TR5
10	Female	12.76	Papillary thyroid carcinoma	Minimal	Negative	TR5
11	Female	8.06	Papillary thyroid carcinoma	Minimal	Positive	TR5
12	Male	10.79	Follicular carcinoma of the thyroid	Significant	Positive	TR3
13	Female	10.96	Follicular carcinoma of the thyroid	Significant	Negative	TR3
14	Female	5.05	Papillary thyroid carcinoma	NO	Positive	TR3
15	Male	12.25	Undifferentiated Thyroid carcinoma	Minimal	Negative	TR5
16	Female	11.82	Papillary thyroid carcinoma	Minimal	Negative	TR5
17	Female	9.37	Papillary thyroid carcinoma	Significant	Negative	TR5
18	Male	8	Papillary thyroid carcinoma	Significant	Positive	TR5
19	Female	10.41	Papillary thyroid carcinoma	Minimal	Negative	TR3
20	Female	11.64	Papillary thyroid carcinoma	Minimal	Negative	TR3
21	Male	11.28	Follicular carcinoma of the thyroid	Minimal	Negative	TR3
22	Female	8.77	Follicular carcinoma of the thyroid	Significant	Negative	TR5
23	Female	8.31	Papillary thyroid carcinoma	Significant	Positive	TR5
24	Male	9.01	Papillary thyroid carcinoma	Minimal	Negative	TR5
25	Female	8.61	Papillary thyroid carcinoma	Minimal	Negative	TR5
26	Male	10.62	Papillary thyroid carcinoma	Significant	Negative	TR4
27	Female	7.69	Papillary thyroid carcinoma	Significant	Positive	TR5
28	Male	8.23	Papillary thyroid carcinoma	Significant	Negative	TR5
29	Female	9.58	Follicular carcinoma of the thyroid	NO	Negative	TR3
30	Male	11.15	Follicular carcinoma of the thyroid	NO	Negative	TR4
31	Female	7.86	Undifferentiated Thyroid carcinoma	Minimal	Positive	TR4
32	Female	11.89	Follicular carcinoma of the thyroid	Minimal	Positive	TR3
33	Male	5.72	Papillary thyroid carcinoma	NO	Positive	TR3
34	Female	11.27	Papillary thyroid carcinoma	Significant	Negative	TR3
35	Female	11.81	Papillary thyroid carcinoma	Minimal	Negative	TR3
36	Female	8.53	Papillary thyroid carcinoma	Significant	Positive	TR5
37	Male	11.01	Papillary thyroid carcinoma	Minimal	Negative	TR4
38	Female	5.8	Papillary thyroid carcinoma	Significant	Positive	TR3
39	Female	7.87	Undifferentiated Thyroid carcinoma	Minimal	Positive	TR4
40	Male	9.07	Papillary thyroid carcinoma	Significant	Negative	TR5
41	Female	7.9	Papillary thyroid carcinoma	Minimal	Positive	TR4
42	Female	7.4	Papillary thyroid carcinoma	Significant	Positive	TR4
43	Female	8.52	Papillary thyroid carcinoma	Minimal	Positive	TR5
44	Female	7.34	Papillary thyroid carcinoma	Significant	Positive	TR4
45	Female	9.39	Follicular carcinoma of the thyroid	Minimal	Negative	TR5
46	Female	10.2	Papillary thyroid carcinoma	Significant	Positive	TR3
47	Male	8.92	Papillary thyroid carcinoma	Minimal	Negative	TR5
48	Female	7.04	Papillary thyroid carcinoma	NO	Positive	TR4
49	Female	8.54	Papillary thyroid carcinoma	Minimal	Negative	TR5
50	Female	6.87	Papillary thyroid carcinoma	NO	Positive	TR5
51	Male	11.82	Papillary thyroid carcinoma	Minimal	Negative	TR5
52	Female	8.22	Follicular carcinoma of the thyroid	Significant	Positive	TR5
53	Female	8.7	Papillary thyroid carcinoma	Minimal	Negative	TR5
54	Female	8.05	Papillary thyroid carcinoma	Significant	Negative	TR5
55	Female	11.78	Medullary thyroid carcinoma	Minimal	Negative	TR3
56	Female	10.27	Papillary thyroid carcinoma	NO	Negative	TR4
57	Female	11.36	Papillary thyroid carcinoma	Minimal	Negative	TR3
58	Female	9.71	Undifferentiated Thyroid carcinoma	NO	Negative	TR4
59	Female	12.54	Papillary thyroid carcinoma	Significant	Negative	TR5
60	Male	6.77	Medullary thyroid carcinoma	NO	Positive	TR4
61	Female	9.81	Papillary thyroid carcinoma	NO	Positive	TR4
62	Female	10.6	Papillary thyroid carcinoma	Minimal	Negative	TR3
63	Female	9.78	Papillary thyroid carcinoma	NO	Negative	TR4
64	Female	10.92	Papillary thyroid carcinoma	NO	Negative	TR4
65	Female	10.76	Papillary thyroid carcinoma	Minimal	Negative	TR3
66	Male	8.46	Papillary thyroid carcinoma	Significant	Negative	TR5
67	Female	9.63	Papillary thyroid carcinoma	NO	Negative	TR4
68	Female	10.64	Papillary thyroid carcinoma	NO	Negative	TR4
69	Female	11.25	Medullary thyroid carcinoma	Minimal	Negative	TR3
70	Male	7.67	Papillary thyroid carcinoma	Minimal	Positive	TR4
71	Female	10.76	Papillary thyroid carcinoma	Minimal	Negative	TR4

### *Overexpression of* NRBP2 *reduces activity of TC cells* in vitro

**Figure 2.**
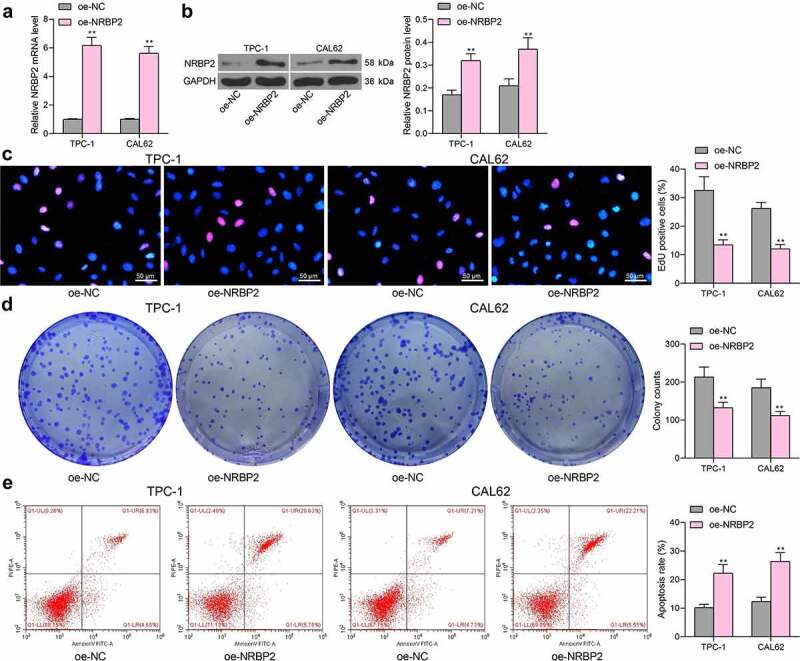
Overexpression of *NRBP2* reduces activity of TC cells *in vitro*. A-B, mRNA (a) and protein (b) levels of NRBP2 in TPC-1 and CAL62 cells after oe-NRBP2 transfection examined by RT-qPCR and western blot assays; (c), DNA replication ability of TPC-1 and CAL62 cells examined by the EdU labeling assay; (d), colony formation ability of TPC-1 and CAL62 cells determined by the colony formation assay; (e), apoptosis rate in TPC-1 and CAL62 cells determined by flow cytometry. Three repetitions were performed. **p < 0.01.

To examine the effect of *NRBP2* on TC cell growth, TPC-1 and CAL62 cells which showed the lowest expression of *NRBP2* were transfected with oe-NRBP2. The successful upregulation was confirmed by RT-qPCR (oe-NC vs. oe-NRBP2 TPC-1: 1.00 vs. 6.17, *p* < 0.0001; CAL62: 1.00 vs. 5.59, *p* < 0.0001) (Figure 2(a)) and western blot assays (oe-NC vs. oe-NRBP2: TPC-1: 0.17 vs. 0.32, *p* < 0.0001; CAL62: 0.21 vs. 0.35, *p* = 0.0016) (Figure 2(b)). Thereafter, the EdU labeling assay demonstrated that the proliferation activity of TPC-1 and CAL62 cells was significantly suppressed (oe-NC vs. oe-NRBP2: TPC-1: 32.28 vs. 13.47, *p* < 0.0001; CAL62: 26.29 vs. 12.09, *p* < 0.0001) (Figure 2(c)). The colony formation assays also suggested that less colonies were formed by cancer cells in the setting of *NRBP2* overexpression when the same number of cells were plated (oe-NC vs. oe-NRBP2: TPC-1: 213.41 vs. 132.58, *p* = 0.0007; CAL62 = 185.27 vs. 18.31, *p* = 0.0013) (Figure 2(d)). Moreover, the flow cytometry results indicated that overexpression of *NRBP2* increased the apoptosis rate in TPC-1 and CAL62 cells (oe-NC vs. oe-NRBP2: TPC-1: 10.26 vs. 21.27, *p* = 0.0002; CAL62: = 12.58 vs. 25.43, *p* < 0.0001) (Figure 2(e)).

### NRBP2 *reduces expression of TME markers and angiogenesis in TC cells*

**Figure 3.**
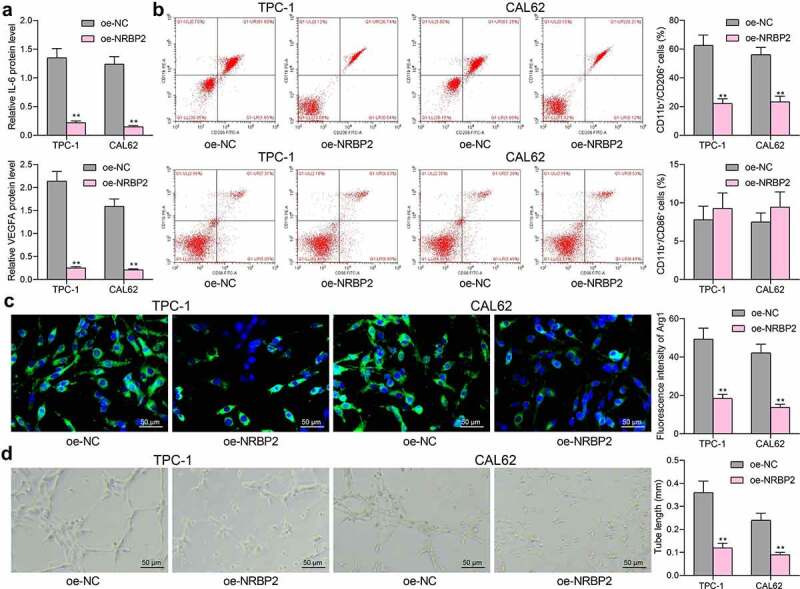
*NRBP2* reduces expression of TME markers and angiogenesis in TC cells. (a), IL-6 and VEGFA concentrations in TPC-1 and CAL62 cell secretions examined using ELISA kits; (b), M1/M2 polarization of macrophages cells co-cultured with TPC-1 and CAL62 cells examined by flow cytometry; (c), expression of the M2-polarized macrophage marker Arg1 in the macrophages determined by immunofluorescence staining; (d), angiogenesis ability of HUVECs co-cultured with TPC-1 and CAL62 cells analyzed by tube formation assay. Three repetitions were performed. **p < 0.01 vs. oe-NC.

To examine the correlation between *NRBP2* and the TME in TC, we first examined the levels of TME markers IL-6 and VEGFA in the supernatant of TPC-1 and CAL62 cells. It was observed that *NRBP2* overexpression significantly decreased the IL-6 and VEGFA levels in the TPC-1 and CAL62 cell secretions (IL-6: oe-NC vs. oe-NRBP2: TPC-1: 1.35 vs. 0.22, *p* < 0.0001; CAL62: 1.24 vs. 0.15, *p* < 0.0001. VEGFA: oe-NC vs. oe-NRBP2: TPC-1: 2.14 vs.0.25, *p* < 0.0001; CAL62: 1.59 vs. 0.22, *p* < 0.0001) (Figure 3(a)). After that, the M0 macrophages were co-cultured with TPC-1 and CAL62 cells, and then the polarization of the macrophages was examined by flow cytometry. It was found that co-culture with cells overexpressing *NRBP2* reduced the portion of M2-polarized macrophages (CD11b^+^ CD206^+^) while the portion of M1-polarized macrophages (CD11b^+^ CD86^+^) was not significantly changed (CD11b^+^ CD206^+^: oe-NC vs. oe-NRBP2: TPC-1: 64.18 vs. 19.76, *p* < 0.0001. CAL62: 57.24 vs. 15.41, *p* < 0.0001. CD11b^+^ CD86^+^: oe-NC vs. oe-NRBP2: TPC-1: 12.64 vs. 15.33, *p* = 0.1056; CAL62: 13.49 vs. 16.09, *p* = 0.1184) (Figure 3(b)). Moreover, the immunofluorescence staining indicated that overexpression of *NRBP2* in cancer cells significantly reduced the fluorescence intensity of Arg1 in the macrophages (oe-NC vs. oe-NRBP2: TPC-1: 49.31 vs. 18.42, *p* < 0.0001; CAL62: 42.16 vs. 13.76, *p* < 0.0001) (Figure 3(c)). Thereafter, transfected TPC-1 and CAL62 cells were co-cultured with HUVECs to examine the effect of *NRBP2* on angiogenesis. It was noteworthy that *NRBP2* upregulation in cancer cells significantly decreased the number of tubes formed by HUVECs (oe-NC vs. oe-NRBP2: TPC-1: 0.36 vs. 0.12, *p* < 0.0001; CAL62: 0.24 vs. 0.09, *p* = 0.0003) (Figure 3(d)).

### NRBP2 *reduces TC tumorigenesis and M2 macrophage infiltration in vivo*

**Figure 4.**
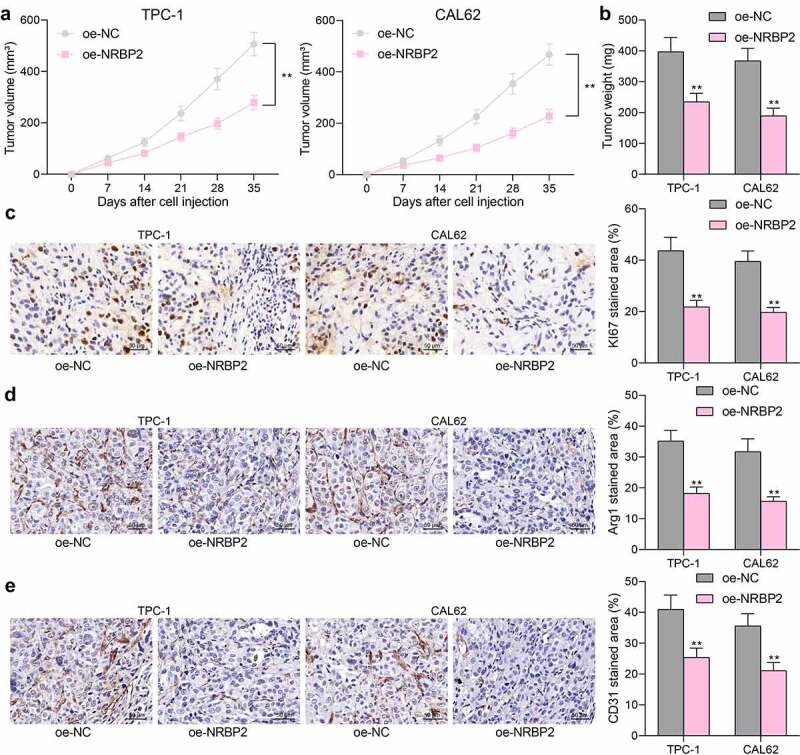
*NRBP2* reduces TC tumorigenesis and M2 macrophage infiltration *in vivo*. (a), growth rate of xenograft tumors formed by TPC-1 and CAL62 cells in nude mice; (b) tumor weight on day 36; (c)-(e), expression of KI67, Arg1 and CD31 in xenograft tumor tissues examined by IHC. In each group, *n* = 6. **p < 0.01 vs. oe-NC.

The role of *NRBP2* in the tumorigenesis of TC *in vivo* was then explored. TPC-1 and CAL62 cells transfected with oe-NRBP2 or oe-NC were injected into mice subcutaneously. The weekly examination to tumor volume suggested that *NRBP2* slowed down the growth rate of xenograft tumors in mice (oe-NC vs. oe-NRBP2: TPC-1: [7 d] 62.52 vs. 44.38, *p* = 0.5851; [14 d]: 124.49 vs. 81.27, *p* = 0.0039; [21 d]: 236.58 vs. 144.62, *p* < 0.0001; [28 d], 370.48 vs. 196.88, (*p* < 0.0001); [35 d]: 506.94 vs. 277.39, *p* < 0.0001; CAL62: [7 d]: 53.61 vs. 36.18, *p* = 0.6389; [14 d]: 131.79 vs. 64.56, *p* < 0.0001; [21 d]: 225.36 vs. 104.08, *p* < 0.0001; [28 d]: 353.64 vs. 161.17, *p* < 0.0001; [35 d]: 466.37 vs. 227.64, *p* < 0.0001) (Figure 4(a)) and decreased the tumor weight on day 36 after animal euthanasia (oe-NC vs. oe-NRBP2: TPC-1: 397.26 vs. 234.97, *p* < 0.0001; CAL62: 367.54 vs. 189.48, *p* < 0.0001) (Figure 4(b)). IHC of tumor tissues showed that *NRBP2* overexpression significantly reduced the positive staining of KI67 in the tumor tissues (oe-NC vs. oe-NRBP2: TPC-1: 43.68 vs. 21.82, *p* < 0.0001; CAL62: 39.51 vs. 19.74, *p* < 0.0001) (Figure 4(c)). In addition, the IHC results also suggested that the staining intensity of Arg1 (M2-macrophage marker) and CD31 (angiogenesis marker) in cells was reduced after *NRBP2* overexpression (Arg1: oe-NC vs. oe-NRBP2: TPC-1: 35.19 vs. 18.24, *p* < 0.0001; CAL62: 31.74 vs. 15.69, *p* < 0.0001; CD31: oe-NC vs. oe-NRBP2: TPC-1: 40.97 vs. 25.38, *p* < 0.0001. CAL62: 35.62 vs. 21.09, *p* < 0.0001) (Figure 4(d,e)). These results suggest that *NRBP2* reduces TC tumorigenesis and M2 macrophage infiltration *in vivo*.

### NRBP2 *is negatively regulated by* GATA1

**Figure 5.**
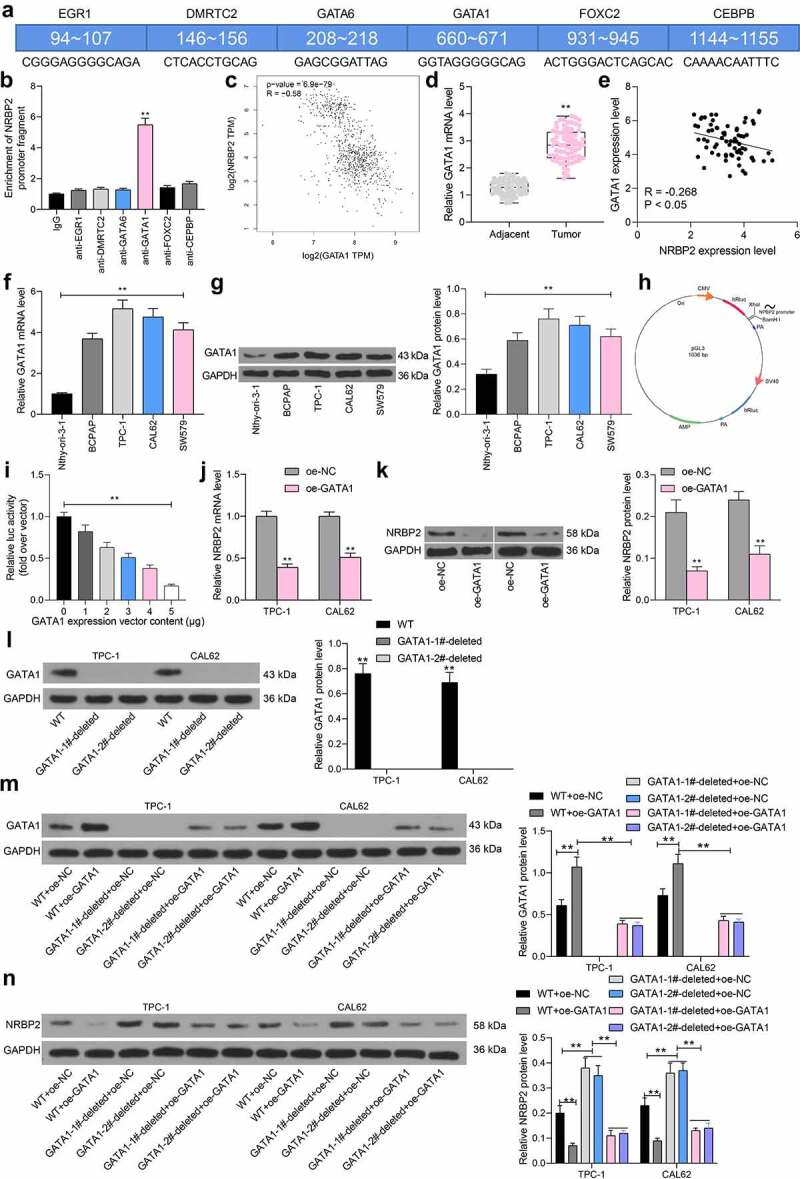
*NRBP2* is negatively regulated by *GATA1*. (a), candidate transcription factors that might bind to the NRBP2 promoter predicted in JASPAR; the numbers in the square frame indicates the binding sites; the gene names are presented on the frames and their corresponding putative binding sequences with *NRBP2* promoter are provided below the frame; (b), binding relationship between *EGR1, DMRTC2, GATA6, GATA1, FOXC2* and *CEBPB* with the promoter of *NRBP2* validated through ChIP-qPCR assays; (c), an inverse correlation between *GATA1* and *NRBP2* expression in TCGA-THCA; (d), GATA1 expression in the tumor and the adjacent tissues from 71 TC patients detected by RT-qPCR; (e), an inverse correlation between *GATA1* and *NRBP2* expression in the collected tumor tissues by Spearman’s correlation analysis; F-G, mRNA (f) and protein (g) levels of NRBP2 in Nthy-ori 3–1 cells and TC cell lines (BCPAP, TPC-1, CAL62 and SW579) detected by RT-qPCR and western blot assays; (h), pGL3 vector containing the *NRBP2* promoter sequence constructed for luciferase assay; I, luciferase activity of the luciferase vector in 293 T cells; J-K, mRNA (j) and protein (k) levels of NRBP2 in TPC-1 and CAL62 cells after oe-GATA1 transfection detected by RT-qPCR; (l), GATA1 protein level in WT or GATA1-deleted cells examined by western blot analysis; (m), GATA1 protein level in cells after further oe-GATA1 transfection examined by western blot analysis; N, NRBP2 protein level in cells examined by western blot analysis. Three repetitions were performed. **p < 0.01.

To determine the upstream regulatory mechanism of *NRBP2*, we first predicted potential transcription factors that have putative binding sites with the *NRBP2* promoter using the JASPAR. Using the prediction score over 14.0 as the threshold, six candidate transcription factors including early growth response 1 (*EGR1)*, DMRT like family C2 (*DMRTC2), GATA6, GATA1*, forkhead box C2 (*FOXC2*) and CCAAT enhancer binding protein beta (*CEBPB*) were predicted to possibly bind with the NRBP2 promoter (Figure 5(a)). Next, ChIP-qPCR assays were conducted to validate the binding relationships. It was observed that anti-GATA1 significantly enriched the *NRBP2* promoter fragments in the immunoprecipitates, but the promoter fragments enriched by anti-EGR1, anti-DMRTC2, anti-GATA6, or anti-FOXC2 showed little difference with those enriched by IgG (IgG vs. anti-GATA1: 1.00 vs. 5.39, *p* < 0.0001) (Figure 5(b)). The data in TCGA-THCA suggested that the *GATA1* expression was inversely correlated with the *NRBP2* expression in normal tissues, cancer tissues, and THCA tissues (Figure 5(c)). RT-qPCR results suggested that the *GATA1* expression was elevated in the collected tumor tissues versus the adjacent tissues (adjacent tissue vs. tumor: 2.14 vs. 4.80, *p* < 0.0001) (Figure 5(d)), which showed an inverse correlation with *NRBP2* expression (r = −0.2674, *p* = 0.0241) (Figure 5(e)). In cells, the GATA1 mRNA and protein levels were significantly elevated in the TC cell compared to the Nthy-ori 3–1 cells (mRNA: Nthy-ori-3-1 vs. BCPAP/TPC-1/CAL62/SW579: 1.00 vs. 3.69/5.16/4.74/4.11, all *p* < 0.0001; protein: Nthy-ori-3-1 vs. BCPAP/TPC-1/CAL62/SW579: 0.32 vs. 0.59/0.76/0.71/0.62, *p* = 0.0028, *p* < 0.0001, *p* = 0.0001, and *p* = 0.0013, respectively) (Figure 5(f,g)). To further validate if *GATA1* regulates the transcription activity of NRBP2, a luciferase vector containing the NRBP2 promoter sequence (Figure 5(h)) was constructed and co-transfected with an ascending series of oe-GATA1 into 293 T cells. The activity of the luciferase vector was significantly decreased as the GATA1 increased (0 μg: 1.00; 1 μg: 0.82; 2 μg: 0.63; 3 μg: 0.51; 4 μg: 0.38; 5 μg: 0.17; 0 μg vs. 1 μg: *p* = 0.0134; 0 μg vs. 2 μg: *p* < 0.0001; 0 μg vs. 3 μg: *p* < 0.0001; 0 μg vs. 4 μg: *p* < 0.0001; 0 μg vs. 5 μg: *p* < 0.0001) (Figure 5(i)). After that, oe-GATA1 was transfected into TPC-1 and CAL62 cells, after which the mRNA and protein levels of NRBP2 were significantly reduced (mRNA: oe-NC vs. oe-GATA1: TPC-1: 1.00 vs. 0.39, *p* < 0.0001; CAL62: 1.00 vs. 0.51, *p* < 0.0001; protein: oe-NC vs. oe-GATA1: TPC-1: 0.21 vs. 0.07, *p* < 0.0001; CAL62:* = *0.24 vs. 0.11, *p* = 0.0001) (Figure 5(j,k)). To identify the transfection efficiency of oe-GATA1, we used the CRISPR-cas9 system to knockout endogenous *GATA1* in TPC-1 and CAL62 cells. It was found that the GATA1 protein was extensively deleted (WT vs. GATA1-1#-deleted: TPC-1: 0.77 vs. 0.00, *p* < 0.0001; CAL62: 0.68 vs. 0.00; *p* < 0.0001; WT vs. GATA1-2#-deleted: TPC-1: 0.77 vs. 0.00, *p* < 0.0001; CAL62: 0.68 vs. 0.00; *p* < 0.0001) (Figure 5(l)). On this basis, it was found that the further administration of oe-GATA1 significantly increased GATA1 level both in WT and GATA1-deleted cells (WT + oe-NC vs. WT + oe-GATA1: T *P*C-1: 0.61 vs. 1.07, *p* < 0.0001; CAL62: 0.73 vs. 1.11, *p* < 0.0001; GATA1-1#-deleted + oe-NC vs. GATA1-1#-deleted + oe-GATA1: T *P*C-1: 0.00 vs. 0.39, *p* < 0.0001; CAL62: 0.00 vs. 0.43, *p* < 0.0001; GATA1-2#-deleted + oe-NC vs. GATA1-1#-deleted + oe-GATA1: T *P*C-1: 0.00 vs. 0.37, *p* < 0.0001; CAL62: 0.00 vs. 0.41, *p* < 0.0001 (Figure 5(m)). Moreover, it was found that the NRBP2 protein expression was significantly elevated in GATA1-deleted cells compared to the WT cells, but the NRBP2 level in both cells was suppressed after oe-GATA-1 transfection (WT + oe-NC vs. GATA1-1#-deleted + oe-NC: TPC-1: 0.20 vs. 0.38, *p* < 0.0001; CAL62: 0.23 vs. 0.36, *p* < 0.0001; WT + oe-NC vs. GATA1-2#-deleted + oe-NC: TPC-1: 0.20 vs. 0.35, *p* < 0.0001; CAL62: 0.23 vs. 0.37, *p* < 0.0001; WT + oe-NC vs. WT + oe-GATA1: TPC-1: 0.20 vs. 0.07, *p < *0.0001; CAL62: 0.23 vs. 0.09, *p < *0.0001; GATA1-1#-deleted + oe-NC vs. GATA1-1#-deleted + oe-GATA1: TPC-1: 0.38 vs. 0.11, *p < *0.0001; CAL62: 0.36 vs. 0.13, *p < *0.0001; GATA1-2#-deleted + oe-NC vs. GATA1-2#-deleted + oe-GATA1: TPC-1: 0.35 vs. 0.12, *p < *0.0001; CAL62: 0.37 vs. 0.14, *p < *0.0001) (Figure 5(n)).

### GATA1 *recruits* HDAC2 *to suppress* NRBP2 *expression*

**Figure 6.**
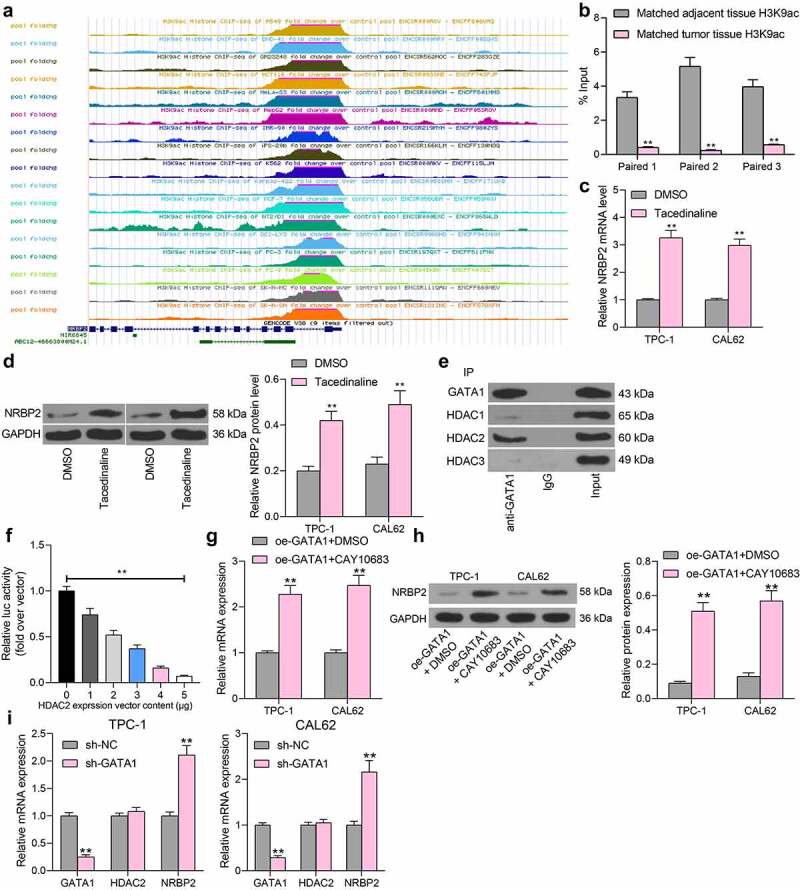
*GATA1* recruits *HDAC2* to suppress *NRBP2* expression. (a), significant H3K9ac in the *NRBP2* promoter predicted in the UCSC browser; (b), H3K9ac level in three pairs of TC tumor tissues and the adjacent tissues examined by the ChIP-qPCR assay; (c)-(d), mRNA (c) and protein (d) levels of NRBP2 in TPC-1 and CAL62 cells after Tacedinaline treatment detected by RT-qPCR and western blot assay; (e), HDACs that can bind to GATA1 in TPC-1 cells validated by the Co-IP assays; input refers to the positive control that confirm the corresponding proteins are expressed in cells; IgG represents the negative control; (f), luciferase activity of the luciferase vector in 293 T cells. Three repetitions were performed; G-H, mRNA (g) and protein (h) expression of NRBP2 in cells after CAY10683 treatment examined by RT-qPCR and western blot analysis; (i), mRNA expression of *GATA1, HDAC2*, and *NRBP2* in cells after sh-GATA1 transfection determined by RT-qPCR. **p < 0.01.

The results above preliminarily suggested that *GATA1* could transcriptionally suppress *NRBP2* expression to induce growth of TC cells and the changes in TME. We then explored whether there are other epigenetic regulations such as DNA methylations and histone modifications that affect *NRBP2* expression. Data in the UCSC browser suggested that a histone modification marker H3K9ac is enriched at the *NRBP2* promoter (Figure 6(a)). Thereafter, we examined the H3K9ac level in three pairs of collected tumor tissues and adjacent tissues using anti-H3K9ac. It was found that the H3K9ac level, namely the histone acetylation level, was significantly reduced in tumor tissues (adjacent tissue vs. tumor: Paired 1: 3.34 vs. 0.41, *p* < 0.0001; Paired 2: 5.16 vs. 0.24. *p* < 0.0001; Paired 3: 3.97 vs. 0.57, *p* < 0.0001) (Figure 6(b)). We then speculated that under pathological conditions of TC, aberrant deacetylation modification might reduce the expression of *NRBP2* and therefore promote the onset and development of TC. To validate this, a HDACs-specific inhibitor Tacedinaline was administrated into TPC-1 and CAL62 cells. It was observed that after the HDAC inhibition, the NRBP2 mRNA and protein levels in cells were significantly enhanced (mRNA: DMSO vs. Tacedinaline: TPC-1: 1.00 vs. 3.26, *p* < 0.0001; CAL62:1.00 vs. 2.98, *p* < 0.0001; protein: DMSO vs Tacedinaline: TPC-1: 0.20 vs. 0.44, *p* = 0.0003; CAL62: 1.00 vs. 2.98, *p* = 0.0002) (Figure 6(c,d)). Linking this to the results in Figure 5, we hypothesized that *GATA1* possibly recruits certain HDACs to the promoter region of *NRBP2* to suppress its expression. Therefore, a Co-IP assay was performed where anti-GATA1 was introduced into TPC-1 and CAL62 cells for IP. After that, the western blot analysis identified HDAC2 expression in the precipitated compounds pulled down by anti-GATA1, whereas no expression of HDAC1 or HDAC3 was detected (Figure 6(e)). To examine the regulation of *HDAC2* on *NRBP2* expression, the luciferase vector containing *NRBP2* promoter sequence was co-transfected with oe-HDAC2 into 293 T cells. Importantly, it was found that oe-HDAC2 also reduced the luciferase activity in cells (0 μg: 1.00; 1 μg: 0.74; 2 μg: 0.52; 3 μg: 0.37; 4 μg: 0.16; 5 μg: 0.07; 0 μg vs. 1 μg: *p* = 0.0001; 0 μg vs. 2 μg: *p* < 0.0001; 0 μg vs. 3 μg: *p* < 0.0001; 0 μg vs. 4 μg: *p* < 0.0001; 0 μg vs. 5 μg: *p* < 0.0001) (Figure 6(f)). Thereafter, the oe-GATA1-transfected TPC-1 and CAL62 cells were further treated with a HDAC2-specific inhibitor CAY10683, after which we found that the expression of NRBP2 was significantly restored (mRNA: oe-GATA1 + DMSO vs. oe-GATA1 + CAY10683 TPC1: 1.00 vs. 2.28, *p* = 0.0002; CAL62: 1.00 vs. 2.36, *p* = 0.0001; protein: oe-GATA1 + DMSO vs. oe-GATA1 + CAY10683: TPC1: 0.09 vs. 0.51, *p* < 0.0001; CAL62 = 0.13 vs. 0.55, *p* < 0.0001) (Figure 6(g,h)). These results further indicate that *HDAC2* is required for *GATA1*-mediated *NRBP2* downregulation. We also induced shRNA silencing of GATA1 in TPC-1 and CAL62 cells. In this setting, it was observed that the *HDAC2* mRNA in cells was not significantly changed, but the *NRBP2* expression was significantly elevated (TPC-1: sh-NC vs. sh-GATA1: GATA1: 1.00 vs. 0.25, *p* < 0.0001; HDAC2: 1.00 vs. 1.08, *p* = 0.7822; NRBP2, 1.00 vs. 2.11, *p* < 0.0001; CAL62: sh-NC vs. sh-GATA1: GATA1: 1.00 vs. 0.29, *p* < 0.0001; HDAC2: 1.00 vs. 1.05, *p* = 0.9402; NRBP2, 1.00 vs. 2.16, *p* < 0.0001) (Figure 6(i)). These results, collectively, suggest that *GATA1* recruits *HDAC2* to the promoter region of *NRBP2* to reduce its expression.

### GATA1 *or* HDAC2 *expression counteracts the inhibitory effect of oe-NRBP2 on TC cells*

**Figure 7.**
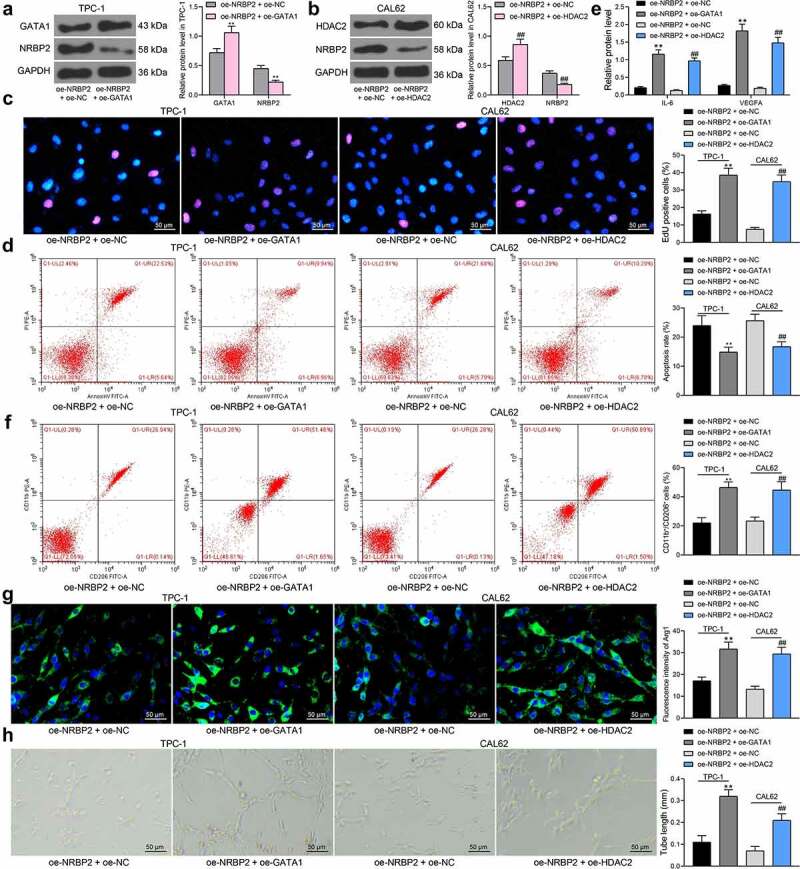
*GATA1* or *HDAC2* overexpression counteracts the inhibitory effect of oe-NRBP2 on TC cells. A-B, protein level of NRBP2 in TPC-1 (a) and CAL62 (b) cells after GATA1 or HDAC2 overexpression, respectively, examined by western blot assays; (c), proliferation activity of TPC-1 and CAL62 cells examined by the EdU labeling assay; (d), apoptosis rate in TPC-1 and CAL62 cells determined by flow cytometry; (e), IL-6 and VEGFA concentrations in TPC-1 and CAL62 cell secretions examined using ELISA kits; (f)-(g), polarization of M2 macrophages cells co-cultured with TPC-1 and CAL62 cells determined by flow cytometry and immunofluorescence staining; (h), angiogenesis ability of HUVECs co-cultured with TPC-1 and CAL62 cells determined by tube formation assay. Three repetitions were performed. **p < 0.01.

The effects of *GATA1* and *HDAC2* on the expression and function of *NRBP2* were further investigated. TPC-1 cells transfected with oe-NRBP2 were further transfected with oe-GATA1, and CAL62 cells with oe-NRBP2 were further transfected with oe-HDAC2. Importantly, the NRBP2 protein expression in cells was decreased following GATA1 or HDAC2 overexpression (TPC-1: oe-NRBP2 + oe-NC vs. oe-NRBP2 + oe-GATA1; GATA1: 0.72 vs. 1.06, *p* = 0.0008; NRBP2: 0.45 vs. 0.22, *p* = 0.0085; CAL62: oe-NRBP2 + oe-NC vs. oe-NRBP2 + oe-GATA1: HDAC2: 0.59 vs. 0.86, *p* = 0.0010; NRBP2: 0.37 vs. 0.18, *p* = 0.0081) (Figure 7(a,b)). Thereafter, the proliferation activity of cells, which was initially inhibited by oe-NRBP2, was recovered by further *GATA1* or *HDAC2* upregulation (oe-NRBP2 + oe-NC vs. oe-NRBP2 + oe-GATA1: 16.27 vs. 38.56, *p* < 0.0001; oe-NRBP2 + oe-NC vs. oe-NRBP2 + oe-HDAC2: 7.49 vs. 34.83, *p* < 0.0001) (Figure 7(c)). Apoptosis rate in TPC-1 and CAL62 cells was significantly decreased after oe-GATA1 or oe-HDAC2 transfection (oe-NRBP2 + oe-NC vs. oe-NRBP2 + oe-GATA1: 23.19 vs. 14.75, *p* = 0.0055; oe-NRBP2 + oe-NC vs. oe-NRBP2 + oe-HDAC2: 26.08 vs. 16.86, *p* = 0.0032) (Figure 7(d)). After that, we further examined the IL-6 and VEGFA levels in cell secretions. The ELISA results showed that the IL-6 and VEGFA levels secreted by TPC-1 or CAL62 cells were significantly elevated after *GATA1* or *HDAC2* overexpression (IL-6: oe-NRBP2 + oe-NC vs. oe-NRBP2 + oe-GATA1: 0.21 vs. 1.16, *p* < 0.0001; oe-NRBP2 + oe-NC vs. oe-NRBP2 + oe-HDAC2: 0.13 vs. 0.97, *p* < 0.0001; VEGFA: oe-NRBP2 + oe-NC vs. oe-NRBP2 + oe-GATA1: 0.27 vs. 1.82, *p* < 0.0001; oe-NRBP2 + oe-NC vs. oe-NRBP2 + oe-HDAC2: 0.19 vs. 1.48, *p* < 0.0001) (Figure 7(e)). In addition, overexpression of *GATA1* or *HDAC2* in TC cells increased the M2 polarization of the co-cultured M0 macrophages (Flow cytometry: oe-NRBP2 + oe-NC vs. oe-NRBP2 + oe-GATA1: 18.43 vs. 47.08, *p* < 0.0001; oe-NRBP2 + oe-NC vs. oe-NRBP2 + oe-HDAC2: 15.87 vs. 39.28, *p* = 0.0001; Immunofluorescence staining: oe-NRBP2 + oe-NC vs. oe-NRBP2 + oe-GATA1: 17.13 vs. 31.67, *p* = 0.0004; oe-NRBP2 + oe-NC vs. oe-NRBP2 + oe-HDAC2: 13.28 vs. 29.43, *p* = 0.0002) (Figure 7(f,g)) and increased the angiogenesis ability of the co-cultured HUVECs (oe-NRBP2 + oe-NC vs. oe-NRBP2 + oe-GATA1: 0.11 vs. 0.32, *p* < 0.0001; oe-NRBP2 + oe-NC vs. oe-NRBP2 + oe-HDAC2: 0.07 vs. 0.21, *p* = 0.0012) (Figure 7(h)).

## Discussion

Angiogenesis is an essential process for the supply of nutrients and oxygen to the rapid growth and dissemination of tumor cells [31]. Aberrant TME plays a critical role in tumor angiogenesis, invasion and metastasis [6,32]. This study reports that *GATA1*- and *HDAC2*-mediated *NRBP2* downregulation induced TME and angiogenesis in TC cells *in vitro* and tumor growth and macrophage infiltration *in vivo*.

Bioinformatics tools including GEO datasets and TCGA-THCA have offered great convenience in the fast identification of hub genes implicated in TC progression [33]. In this work, by analyzing the GSE165724 dataset and TCGA-THCA, we obtained *NRBP2* as a significantly downregulated gene in TC. The poor expression of *NRBP2* was validated in the clinical tissues and TC cell lines. Low *NRBP2* expression was linked to tumor infiltration, lymph node metastasis, and the consequent poor prognosis of patients. *NRBP2* shows a 59% amino acid similarity to *NRBP1* which has been identified to regulate intestinal progenitor cell homeostasis and suppress tumor formation [34]. As for *NRBP2* itself, it has recently been demonstrated as a tumor inhibitor in breast cancer since its poor expression was related to poor prognosis of patients and its high expression limited tumor metastasis [35]. Likewise, *NRBP2* was poorly expressed in medulloblastoma and it reduced survival and growth of tumor cells upon overexpression [36]. In addition, *NRBP2* showed a chemo-sensitizing effect in hepatocellular carcinoma cells [37]. Our experimental results first showed that *NRBP2* weakened proliferation and colony formation abilities of TC cells and increased cell apoptosis.

There is not always a direct correlation between gene alteration and the invasiveness of TC, whereas the altered gene expression may affect the TME to influence the tumor angiogenesis and dissemination [6]. It has been well-recognized that the TME is beneficial for many stages of tumor development from occurrence to metastasis, and it largely affects tumor treatment and the clinical outcome [38]. However, current studies concerning TME of TC are contradictory. For instance, tumor-infiltrating lymphocytes (TILs) were reported to be linked to extra-thyroidal extension [39], but the following researches of TILs did not have a significant positive role in TC [40]. Elevated CD8^+^ T cell tumor infiltration in patients with differentiated PTC was correlated with increased disease-free survival [41]. However, infiltration of CD8^+^ T cell has also been observed to predict relapse in PTC [42]. Among the immune regulators, tumor-associated macrophages (TAMs), mostly the immunosuppressive M2 types, are generally playing tumor enhancing roles in PTC and are present in more aggressive tumor types [41,43,44]. The M2 macrophages are closely correlated with angiogenesis and lymph angiogenesis in cancer [45]. Importantly, our experiments suggested that *NRBP2* overexpression significantly decreased the expression of TME biomarkers IL-6 and VEGFA [46] in the TPC-1 and CAL62 cells and, and overexpression of *NRBP2* in cells reduced the M2 polarization of the co-cultured macrophages. In addition, it was found that the angiogenesis ability of HUVECs was reduced when cultured in an *NRBP2*-overexpressing condition. *In vivo*, overexpression of *NRBP2* also reduced weight and volume of and the TAM infiltration in the xenograft tumors. These results revealed a tumor-suppressing role of NRBP2 in TC by weakening TME and angiogenesis.

Our subsequent integrated bioinformatic analyses and ChIP-qPCR assays suggested that *GATA1* negatively regulated NRBP2 transcription. The tumor-promoting role of GATA1 has been witnessed in human malignancies such as ovarian cancer [13] and colorectal cancer [14] by promoting cell proliferation and invasiveness. *GATA1* is a critical factor in erythropoiesis, while little has been concerned about its involvement in TME. As for angiogenesis, *GATA1* has been reported to interact with the histone methyltransferase SET7 to trigger VEGF-induced angiogenesis in breast cancer [16]. Importantly, by using the UCSC browser concerning the potential epigenetic regulations, we predicated that there is a significant H3K9ac modification near the *NRBP2* promoter. However, the H3K9ac modification, which is usually correlated with gene activation, was reduced in the NRBP2 promoter in TC tissues. In addition, we found that treatment with a HDAC inhibitor Tacedinaline restored the H3K9ac level in the *NRBP2* promoter in TPC-1 and CAL62 cells, and treatment with the HDAC2-specific inhibitor CAY10683 restored the NRBP2 levels in cells suppressed by oe-GATA1, indicating that *HDAC2* is required for *GATA1*-mediated *NRBP2* downregulation. The transcriptional co-factor friend of *GATA1* was found to recruit histone deacetylase *NuRD* to the mast cell gene promoter [47]. We then surmised that *GATA1* may recruit specific *HDACs* to the *NRBP2* promoter to induce transcriptional repression via deacetylation of H3K9ac. Importantly, the Co-IP, western blot, and dual immunofluorescence staining assays confirmed a binding relationship between GATA1 and HDAC2 in the nucleus of TC cells. *HDAC2* is a widely investigated *HDAC* which locates in nucleus and can exert functions alone [48]. *HDAC2* regulates gene transcription by deacetylating the N-terminal tails of the core histones, leading to more condensed chromatin state and reduced transcriptional activity [49]. A study by Li *et al*. suggested that a T-box transcription factor *TBX3* recruits *HDAC1* and *HDAC2* to transcriptionally suppress expression of p57 to promote proliferation of PTC cells [50]. Here, we confirmed that overexpression of *GATA1* or *HDAC2* blocked the roles of *NRBP2* and restored proliferation of PTC cells. Inhibition of *HDACs* has been reported to induce M2 polarization of macrophages in nitrogen mustard-induced lung injury [51]. Here, we confirmed that overexpression of *GATA1* or *HDAC1* strengthened the TME, induced M2 polarizations of macrophages and induced angiogenesis of HUVECs co-cultured with the PTC cells.

## Conclusion

**Figure 8.**
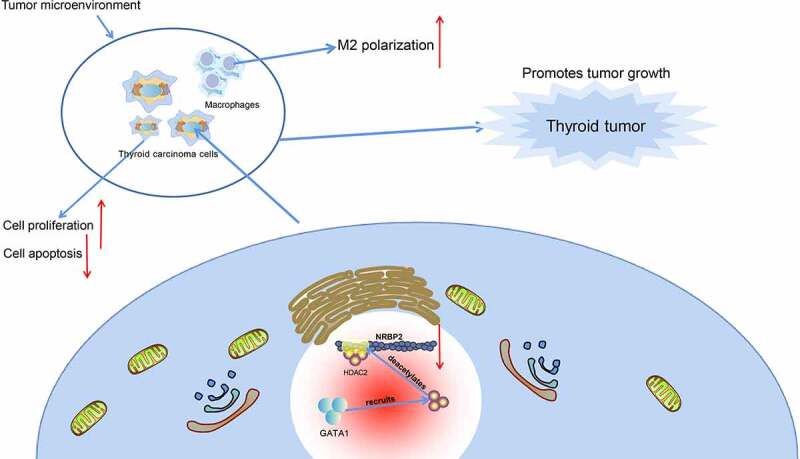
Graphical abstract. In TC, *GATA1* recruits *HDAC2* to the promoter region of *NRBP2* to induce *NRBP2* transcriptional suppression by deacetylation, which leads to increased M2 polarization of macrophages as well as increased angiogenesis, proliferation, and tumor development.

In conclusion, this study reports that *GATA1* can recruit *HDAC2* to the *NRBP2* promoter to induce its transcriptional suppression, which leads to M2 polarization of macrophages and tumor angiogenesis and development (Figure 8). *GATA1* and *HDAC2* may serve as potential therapeutic targets for TC, though more intensive pre-clinical researches are required. Hopefully we will see more new findings in TME in TC as this field is developing rapidly.

## Data Availability

All the data generated or analyzed during this study are included in this published article.

## References

[1] Bray F, Ferlay J, Soerjomataram I, et al. Global cancer statistics 2018: GLOBOCAN estimates of incidence and mortality worldwide for 36 cancers in 185 countries. CA Cancer J Clin. 2018;68:394–424. 30207593 10.3322/caac.21492

[2] Staniforth JUL, Erdirimanne S, Eslick GD. Thyroid carcinoma in Graves’ disease: a meta-analysis. Int J Surg. 2016;27:118–125. 26626367 10.1016/j.ijsu.2015.11.027

[3] Baloch ZW, LiVolsi VA. Special types of thyroid carcinoma. Histopathology. 2018;72:40–52. 29239042 10.1111/his.13348

[4] Deeken-Draisey A, Yang GY, Gao J, et al. Anaplastic thyroid carcinoma: an epidemiologic, histologic, immunohistochemical, and molecular single-institution study. Hum Pathol. 2018;82:140–148. 30075157 10.1016/j.humpath.2018.07.027

[5] Wang Y, Zong H, Zhou H. Circular RNA circ_0062389 modulates papillary thyroid carcinoma progression via the miR-1179/high mobility group box 1 axis. Bioengineered. 2021;12:1484–1494. 33926347 10.1080/21655979.2021.1914470 PMC8806330

[6] Bergdorf K, Ferguson DC, Mehrad M, et al. Papillary thyroid carcinoma behavior: clues in the tumor microenvironment. Endocr Relat Cancer. 2019;26:601–614. 30965283 10.1530/ERC-19-0074 PMC8279427

[7] Shi L, Zhao SM, Luo Y, et al. MiR-375: a prospective regulator in medullary thyroid cancer based on microarray data and bioinformatics analyses. Pathol Res Pract. 2017;213:1344–1354. 29033189 10.1016/j.prp.2017.09.024

[8] Wang S, Liu F, Wang Y, et al. Integrated analysis of 34 microarray datasets reveals CBX3 as a diagnostic and prognostic biomarker in glioblastoma. J Transl Med. 2019;17:179. 31138312 10.1186/s12967-019-1930-3 PMC6540543

[9] De Langhe S, Haataja L, Senadheera D, et al. Interaction of the small GTPase Rac3 with NRBP, a protein with a kinase-homology domain. Int J Mol Med. 2002;9:451–459. 11956649

[10] Wang H, Sun X, Luo Y, et al. Adapter protein NRBP associates with Jab1 and negatively regulates AP-1 activity. FEBS Lett. 2006;580:6015–6021. 17052710 10.1016/j.febslet.2006.10.002

[11] Yeh SJ, Lin CY, Li CW, et al. Systems biology approaches to investigate genetic and epigenetic molecular progression mechanisms for identifying gene expression signatures in papillary thyroid cancer. Int J Mol Sci. 2019;20:2536. 10.3390/ijms20102536 PMC6566633 31126066

[12] Gutierrez L, Caballero N, Fernandez-Calleja L, et al. Regulation of GATA1 levels in erythropoiesis. IUBMB Life. 2020;72:89–105. 31769197 10.1002/iub.2192

[13] Liu Z, Zhu Y, Li F, et al. GATA1-regulated JAG1 promotes ovarian cancer progression by activating Notch signal pathway. Protoplasma. 2020;257:901–910. 31897811 10.1007/s00709-019-01477-w

[14] Yu J, Liu M, Liu H, et al. GATA1 promotes colorectal cancer cell proliferation, migration and invasion via activating AKT signaling pathway. Mol Cell Biochem. 2019;457:191–199. 31069596 10.1007/s11010-019-03523-w

[15] Audia JE, Campbell RM. Histone modifications and cancer. Cold Spring Harb Perspect Biol. 2016;8:a019521. 27037415 10.1101/cshperspect.a019521 PMC4817802

[16] Zhang Y, Liu J, Lin J, et al. The transcription factor GATA1 and the histone methyltransferase SET7 interact to promote VEGF-mediated angiogenesis and tumor growth and predict clinical outcome of breast cancer. Oncotarget. 2016;7:9859–9875. 26848522 10.18632/oncotarget.7126 PMC4891089

[17] Webber LP, Wagner VP, Curra M, et al. Hypoacetylation of acetyl-histone H3 (H3K9ac) as marker of poor prognosis in oral cancer. Histopathology. 2017;71:278–286. 28326594 10.1111/his.13218

[18] Tessler FN, Middleton WD, Grant EG, et al. ACR Thyroid Imaging, Reporting and Data System (TI-RADS): white paper of the ACR TI-RADS committee. J Am Coll Radiol. 2017;14:587–595. 28372962 10.1016/j.jacr.2017.01.046

[19] Jin Y, Chen L, Li L, et al. SNAI2 promotes the development of ovarian cancer through regulating ferroptosis. Bioengineered. 2022;13:6451–6463. 35220872 10.1080/21655979.2021.2024319 PMC8974033

[20] Ding C, Shi T, Wu G, et al. The anti-cancer role of microRNA-143 in papillary thyroid carcinoma by targeting high mobility group AT-hook 2. Bioengineered. 2022;13:6629–6640. 35213273 10.1080/21655979.2022.2044277 PMC8973723

[21] Xu Y, Gao F, Zhang J, et al. Fibroblast growth factor receptor 2 promotes the proliferation, migration, and invasion of ectopic stromal cells via activation of extracellular-signal-regulated kinase signaling pathway in endometriosis. Bioengineered. 2022;13:8360–8371. 35311468 10.1080/21655979.2022.2054207 PMC9161834

[22] Zhu G, Cheng Z, Lin C, et al. MyD88 regulates LPS-induced NF-kB/MAPK cytokines and promotes inflammation and malignancy in colorectal cancer cells. Cancer Genomics Proteomics. 2019;16:409–419. 31659096 10.21873/cgp.20145 PMC6885359

[23] An Y, Yang Q. MiR-21 modulates the polarization of macrophages and increases the effects of M2 macrophages on promoting the chemoresistance of ovarian cancer. Life Sci. 2020;242:117162. 31837336 10.1016/j.lfs.2019.117162

[24] Cai D, Chen C, Su Y, et al. LRG1 in pancreatic cancer cells promotes inflammatory factor synthesis and the angiogenesis of HUVECs by activating VEGFR signaling. J Gastrointest Oncol. 2022;13:400–412. 35284128 10.21037/jgo-21-910 PMC8899736

[25] Ye M, Gao R, Chen S, et al. Downregulation of MEG3 and upregulation of EZH2 cooperatively promote neuroblastoma progression. J Cell Mol Med. 2022;26:2377–2391. 35257481 10.1111/jcmm.17258 PMC8995459

[26] Song H, Chen S, Zhang T, et al. Integrated strategies of diverse feature selection methods identify aging-based reliable gene signatures for ischemic cardiomyopathy. Front Mol Biosci. 2022;9:805235. 35300115 10.3389/fmolb.2022.805235 PMC8921505

[27] Xu S, Jiang Y, Duan Y. hsa_circ_0077837 alleviated the malignancy of non-small cell lung cancer by regulating the miR-1178-3p/APITD1 axis. J Oncol. 2022;2022:3902832. 35310916 10.1155/2022/3902832 PMC8926487

[28] Qian S, Han X, Sha X, et al. Aqueous extract of cimicifuga dahurica reprogramming macrophage polarization by activating TLR4-NF-kappaB signaling pathway. J Inflamm Res. 2022;15:1027–1046. 35210810 10.2147/JIR.S345497 PMC8858003

[29] Guo R, Liang Y, Zou B, et al. The histone acetyltransferase MOF regulates SIRT1 expression to suppress renal cell carcinoma progression. Front Oncol. 2022;12:842967. 35252011 10.3389/fonc.2022.842967 PMC8888902

[30] Mao X, Lei H, Yi T, et al. Lipid reprogramming induced by the TFEB-ERRalpha axis enhanced membrane fluidity to promote EC progression. J Exp Clin Cancer Res. 2022;41:28. 35045880 10.1186/s13046-021-02211-2 PMC8767755

[31] Folkman J. Angiogenesis. Annu Rev Med. 2006;57:1–18. 16409133 10.1146/annurev.med.57.121304.131306

[32] Min AKT, Mimura K, Nakajima S, et al. Therapeutic potential of anti-VEGF receptor 2 therapy targeting for M2-tumor-associated macrophages in colorectal cancer. Cancer Immunol Immunother. 2021;70:289–298. 32705303 10.1007/s00262-020-02676-8 PMC10991089

[33] Pan Y, Wu L, He S, et al. Identification of hub genes in thyroid carcinoma to predict prognosis by integrated bioinformatics analysis. Bioengineered. 2021;12:2928–2940. 34167437 10.1080/21655979.2021.1940615 PMC8806580

[34] Wilson CH, Crombie C, van der Weyden L, et al. Nuclear receptor binding protein 1 regulates intestinal progenitor cell homeostasis and tumour formation. EMBO J. 2012;31:2486–2497. 22510880 10.1038/emboj.2012.91 PMC3365428

[35] Li Z, Liu B, Li C, et al. NRBP2 functions as a tumor suppressor and inhibits epithelial-to-mesenchymal transition in breast cancer. Front Oncol. 2021;11:634026. 33816275 10.3389/fonc.2021.634026 PMC8012753

[36] Xiong A, Roy A, Spyrou A, et al. Nuclear receptor binding protein 2 is downregulated in medulloblastoma, and reduces tumor cell survival upon overexpression. Cancers (Basel). 2020;12:1483. 10.3390/cancers12061483 PMC7352854 32517178

[37] Zhang L, Ge C, Zhao F, et al. NRBP2 overexpression increases the chemosensitivity of hepatocellular carcinoma cells via akt signaling. Cancer Res. 2016;76:7059–7071. 27634758 10.1158/0008-5472.CAN-16-0937

[38] Wu P, Shi J, Sun W, et al. The prognostic value of plasma complement factor B (CFB) in thyroid carcinoma. Bioengineered. 2021;12:12854–12866. 34898340 10.1080/21655979.2021.2005745 PMC8810132

[39] Matsubayashi S, Kawai K, Matsumoto Y, et al. The correlation between papillary thyroid carcinoma and lymphocytic infiltration in the thyroid gland. J Clin Endocrinol Metab. 1995;80:3421–3424. 8530576 10.1210/jcem.80.12.8530576

[40] Lundgren CI, Hall P, Dickman PW, et al. Clinically significant prognostic factors for differentiated thyroid carcinoma: a population-based, nested case-control study. Cancer. 2006;106:524–531. 16369995 10.1002/cncr.21653

[41] Cunha LL, Morari EC, Guihen AC, et al. Infiltration of a mixture of immune cells may be related to good prognosis in patients with differentiated thyroid carcinoma. Clin Endocrinol (Oxf). 2012;77:918–925. 22738343 10.1111/j.1365-2265.2012.04482.x

[42] Cunha LL, Marcello MA, Nonogaki S, et al. CD8+ tumour-infiltrating lymphocytes and COX2 expression may predict relapse in differentiated thyroid cancer. Clin Endocrinol (Oxf). 2015;83:246–253. 25130519 10.1111/cen.12586

[43] Galdiero MR, Varricchi G, Marone G. The immune network in thyroid cancer. Oncoimmunology. 2016;5:e1168556. 27471646 10.1080/2162402X.2016.1168556 PMC4938375

[44] Kim BH. The expression of tumor-associated macrophages in papillary thyroid carcinoma. Endocrinol Metab (Seoul). 2013;28:178–179. 24396676 10.3803/EnM.2013.28.3.178 PMC3811693

[45] Corliss BA, Azimi MS, Munson JM, et al. Macrophages: an inflammatory link between angiogenesis and lymphangiogenesis. Microcirculation. 2016;23:95–121. 26614117 10.1111/micc.12259 PMC4744134

[46] Wang YC, Dai Y, Xu GL, et al. Association between EphA1 and tumor microenvironment in gastric carcinoma and its clinical significance. Med Sci Monit. 2020;26:e923409. 32218416 10.12659/MSM.923409 PMC7133419

[47] Gao Z, Huang Z, Olivey HE, et al. FOG-1-mediated recruitment of NuRD is required for cell lineage re-enforcement during haematopoiesis. EMBO J. 2010;29:457–468. 20010697 10.1038/emboj.2009.368 PMC2824465

[48] Tian P, Zhu Y, Zhang C, et al. Ras-ERK1/2 signaling contributes to the development of colorectal cancer via regulating H3K9ac. BMC Cancer. 2018;18:1286. 30577849 10.1186/s12885-018-5199-3 PMC6303919

[49] Thambyrajah R, Fadlullah MZH, Proffitt M, et al. HDAC1 and HDAC2 modulate TGF-beta signaling during endothelial-to-hematopoietic transition. Stem Cell Reports. 2018;10:1369–1383. 29641990 10.1016/j.stemcr.2018.03.011 PMC5998800

[50] Li X, Ruan X, Zhang P, et al. TBX3 promotes proliferation of papillary thyroid carcinoma cells through facilitating PRC2-mediated p57(KIP2) repression. Oncogene. 2018;37:2773–2792. 29511350 10.1038/s41388-017-0090-2

[51] Venosa A, Gow JG, Hall L, et al. Regulation of nitrogen mustard-induced lung macrophage activation by valproic acid, a histone deacetylase inhibitor. Toxicol Sci. 2017;157:222–234. 28184907 10.1093/toxsci/kfx032 PMC6075217

